# Adverse Effects of Ramadan Fasting in a Girl with Salt-Losing Congenital Adrenal Hyperplasia

**DOI:** 10.1155/2020/6688927

**Published:** 2020-12-29

**Authors:** Valeria Calcaterra, Francesco Bassanese, Andrea Martina Clemente, Rossella Amariti, Corrado Regalbuto, Anna Sala, Gian Vincenzo Zuccotti

**Affiliations:** ^1^Pediatric and Adolescent Unit, Department of Internal Medicine University of Pavia, Pavia, Italy; ^2^Department of Pediatrics, Children' Hospital “Vittore Buzzi”, Milan, Italy; ^3^Pediatric Unit, Department of the Mother and Child Health Fondazione IRCCS Policlinico San Matteo and University of Pavia, Pavia, Italy; ^4^Department of Biomedical and Clinical Science “L. Sacco”, University of Milano, Milano, Italy

## Abstract

**Objective:**

Congenital adrenal hyperplasia (CAH) is the most common cause of adrenal insufficiency in pediatrics. Chronic glucocorticoid replacement is the mainstay of treatment in the classic forms of CAH, and mineralocorticoid replacement therapy is mandatory in the salt-wasting form. Fasting is a mild stressor, which can expose to dehydration, hypotension, hypoglycemia, and acute adrenal crisis in patients with adrenal insufficiency.

**Case:**

We report the case of an adolescent affected by the classic form with salt-losing CAH, who observed Ramadan for 30 days, without individualized therapeutic management plan. After Ramadan, a dramatic increase of ACTH level (1081 pg/ml, n.v. 6–57), reduced cortisolemia, tendency to hypotension, and weight loss were recorded. She experienced insomnia, intense thirst, asthenia, and headache. The symptoms disappeared restarting the previous therapy schedule and increasing the total hydrocortisone daily dose with progressive restoring of hormonal control.

**Conclusion:**

Our case confirms that patients with CAH are vulnerable, especially during fasting in Ramadan, with a higher risk of acute adrenal crisis. CAH patients should reform and individualize their treatment plan and be submitted to careful monitoring.

## 1. Introduction

Congenital adrenal hyperplasia (CAH) is the most common cause of adrenal insufficiency in pediatrics [1]. CAH comprises a family of autosomal recessive disorders that impair adrenal steroidogenesis; the main form is due to 21-hydroxylase deficiency (21-OHD) associated with mutations in the 21-hydroxylase gene (6p21). In the case of 21-OHD, the phenotypic spectrum includes salt wasting, simple virilizing, and late onset form. The “classic form” includes salt wasting and simple virilizing forms. The “late-onset or nonclassic form” includes milder forms [1]. However, this classification system is somewhat artificial because of disease severity due to a continuum based on residual enzyme activity [2]. Chronic glucocorticoid replacement is the mainstay of treatment in the classic forms of CAH. Mineralocorticoid replacement is also necessary in salt-wasting form. Patients in glucocorticoid treatment must double or triple hydrocortisone dose during intercurrent illness (fever and gastrointestinal illness), stressful life events, surgery, or trauma in order to prevent adrenal crisis [3].

Fasting is usually considered as a mild stressor. It can expose to dehydration, hypotension, hypoglycemia, and acute adrenal crisis in patients with adrenal insufficiency. Ramadan fasting is the fourth pillar of Islam. During this holy month, Muslims must abstain from drinking, eating, and even taking oral medications, from sunrise to sunset. This practice is request to all the Muslims, beginning from adolescence, excluding pregnant women and ill subjects in which Ramadan can be dangerous [4]. Nevertheless, some people with chronic conditions still want to adhere fasting during Ramadan.

Here, we report the case of an adolescent affected by the classic form with salt-losing CAH, who observed Ramadan for 30 days, without individualized therapeutic management plan.

## 2. Case Report

The patient is a 13-year-old girl, third child of nonconsanguineous parents, born at the end of a normal course pregnancy, by eutocic birth. Increased 17-OHP level (252 nmol/L) was found at Guthrie neonatal screening and confirmed by a second test (440 nmol/L). Classical CAH form with salt losing was diagnosed, and replacement therapy with hydrocortisone, acetate fludrocortisone, and NaCl oral supplementation was therefore started. During infancy and childhood, there were no remarkable events in her medical history except for episodes of febrile convulsions; secondary enuresis at the age of 6 was treated with oxybutynin for 6 months. Growth and neuropsycomotory development were normal. Menarche occurred at 12 years. During the follow-up (10 years), therapeutic adjustment in medications was related to weight gain and stress periods (fever). Despite the adherence of the therapy was not always optimal, major complications never occurred. The data at the last checkup visit (4 months before Ramadan beginning) are reported in Table 1. Without consulting our clinic or agreeing on therapeutic plans, the girl begins Ramadan, which she completes after a total of 30 days. During Ramadan, the girl observed fasting from sunrise to sunset, having dinner at 9 pm, snacks at midnight, and abundant breakfast at 4 am. Consequently, the patient modified therapy schedule as reported in Table 1. At the periodic endocrinological evaluation, which almost coincided with the end of Ramadan, we found a dramatic increase of ACTH level (1081 pg/ml, n.v. 6–57), reduced cortisolemia (5 mcg/dl, n.v. 4.3–22.2), tendency to hypotension, and important weight loss (−12.5% of body weight) (Table 1).

From the anamnestic collection, it emerged that, during the fasting period, the girl presented some difficulty falling asleep in the first days of Ramadan. She complained intense thirst and strong predilection for salty foods, mainly due to the fast and the saline loss, compensated only during the night. She also reported onset of asthenia and episodes of daytime headache. During this control visit, we prescribed restoring the previous therapy schedule and increasing the total hydrocortisone daily dose to 30 mg. One month after this therapeutic change, the ACTH level was diminished, still indicative of poor control of the CAH, but the symptoms had disappeared, and body weight increased by 2 kg. Four months later, at the end of Ramadan, ACTH hematic level further reduced, and the cortisolemia was within normal ranges. Progressive restoring of hormonal control was reached (Table 1).

Finally, the patient and the family discussed on the seriousness of the decision taken and on the risks that the girl could have faced.

## 3. Discussion

Fasting during Ramadan is a stressful period, in which food and fluid ingestion are restricted from sunrise to sunset for a complete month, each year. As in our patient, during Ramadan, the disruption of feeding and sleep schedules in lack of an adequate adaptation in therapeutic management influences the stress system response and leads to the disruption of the cortisol circadian pattern, with lower levels in the morning and higher in the evening.

The dysregulation of the cortisol circadian rhythm, which acts as a regulator between central and peripheral clocks in different tissues, can have metabolic adverse effects. In patients with adrenal insufficiency, particularly in subjects with classic CAH, Ramadan fasting exposes to the risk of hypoglycemia and dehydration, and this practice could be fatal [5]. Fatigue and asthenia, as noted in this case, can occur as symptoms of the hypoglycemic events. The pathogenic mechanism underlying hypoglycemia is accounted to low levels of cortisolemia, reduction of hepatic gluconeogenesis, and increased sensitivity to insulin, due to relationship between the cortisol rhythm and the insulin resistance rhythm [5]. Fasting shifts from carbohydrates to fatty acid metabolism, using ketones as a major cellular fuel source for the body. In lack of exogenous glucose, counterregulatory hormones, such as glucagon, epinephrine, cortisol, and growth hormone, stimulate hepatic glycogenolysis and gluconeogenesis to maintain glycemic homeostasis. A prevalent use of fatty acids from adipose tissue may also induce body weight loss. Although this reduction may seem beneficial, an exacerbation of this mechanism could lead to an acute adrenal crisis [5].

The distortion of adrenal hormones during Ramadan fasting is also attributed to changes in sleep and activity timing. Sleep rhythm and changes in metabolic status are closely and reciprocally bound: sleeping rhythm alteration can cause distortion of adrenal hormone excretion, and alteration of these later can affect the circadian rhythm, leading to sleep alteration. In our case, sleeplessness could be related to higher levels of cortisol in the evening, due to the assumption of two doses of hydrocortisone close to bedtime, when maximal glucocorticoid sensitivity of peripheral tissues is reported [4, 6]. The observed metabolic impairments during Ramadan practice highlight the need of an individualized therapeutic management plan; intramuscular hydrocortisone [7], subcutaneous glucocorticoids pumps [8], and use of long acting steroids such as dexamethasone and prednisone instead of hydrocortisone [6] could be considered as therapeutic options to adhere Ramadan.

Furthermore, they might adequate their diet habits (avoiding sugars, processed starches, caffeine, and other stimulant drugs, supplementing salt, and drinking nonsugar-laden liquids) and physical activity (increasing resting in fasting and hotter hours). Education of these patients and caregivers is mandatory to recognize warning symptoms of adrenal insufficiency, such as asthenia, nausea, and/or vomiting [9].

In conclusion, our case confirms that patients with CAH are vulnerable, especially during fasting in Ramadan, with a higher risk of acute adrenal crisis. CAH patients should reform and individualize their treatment plan and be submitted to careful monitoring.

## Figures and Tables

**Table 1:** Hormonal panel (8 am), blood pressure, body weight, and therapy of our patient before, during, and after Ramadan.

	Before Ramadan	During Ramadan	3 days after Ramadan	1 month after Ramadan	8 months after Ramadan
ACTH (pg/ml, n.v. 6–57)	236	na	1081	875	16.3
17-OHP (ng/ml, n.v. 0.1–1)	3.7	na	2.4	na	0.7
Cortisol (mcg/dl, n.v. 4.3–22.2)	20.6	na	5	13.2	11.6
Blood pressure (mmHg)	125/80	na	90/60	120/80	110/60
Weight (kg)	62	na	54	56	59
Hydrocortisone	8 am: 10 mg	4 am: 10 mg	8 am: 10 mg	8 am: 10 mg	8 am: 10 mg
	1 pm: 10 mg	9 pm: 10 mg	1 pm: 10 mg	1 pm: 10 mg	1 pm: 10 mg
	8 pm: 5 mg	Midnight: 5 mg	8 pm: 10 mg	8 pm: 10 mg	8 pm: 10 mg
Fludrocortisone	1 pm: 0.1 mg	9 pm: 0.1 mg	1 pm: 0.1 mg	1 pm: 0.1 mg	1 pm: 0.1 mg

na = not available.
